# Alcohol Use and the Risk of Developing Alzheimer’s Disease

**Published:** 2001

**Authors:** Suzanne L. Tyas

**Affiliations:** Suzanne L. Tyas, Ph.D., is an assistant professor in the Ph.D. program in gerontology at the Sanders-Brown Center on Aging and at the Kentucky School of Public Health, University of Kentucky, Lexington, Kentucky

**Keywords:** Alzheimer’s disease, chronic AODE (effects of alcohol or other drugs), AODR (alcohol or other drug-related) dementia, risk factors, cognitive and memory disorder, cholinergic receptors, drug interaction, alcoholic beverage, nicotine, diagnostic criteria, disease course, survey of research

## Abstract

Some of the detrimental effects of heavy alcohol use on brain function are similar to those observed with Alzheimer’s disease (AD). Although alcohol use may be a risk factor for AD, it is difficult to study this relationship because of similarities between alcoholic dementia and AD and because standard diagnostic criteria for alcoholic dementia have not yet been developed. Similar biological mechanisms may be involved in the effects of AD and alcohol abuse on the brain. Epidemiologic studies have investigated the relationship between alcohol use and AD but have not provided strong evidence to suggest that alcohol use influences the risk of developing AD. Further research is needed before the effect of alcohol use on AD is understood fully.

Alzheimer’s disease (AD) is a degenerative brain disorder characterized by a progressive loss of memory and other detrimental cognitive changes as well as lowered life expectancy (Morris 1999). It is the leading cause of dementia in the United States. Aside from the substantial personal costs, AD is a major economic burden on health care and social services (Ernst and Hay 1994; Leon et al. 1998). Estimates of the number of people with AD in the United States in 1997 ranged from 1 million to more than 4 million, and these figures are expected to quadruple within 50 years unless effective interventions are developed (Brookmeyer et al. 1998). The risk of AD increases exponentially with age (Kawas and Katzman 1999); consequently, as the population ages, the importance of AD as a public health concern grows, as does the need for research on the cause of AD and on strategies for its prevention and treatment.

Studying factors that influence the risk of developing AD may lead to the identification of those at high risk for developing it, strategies for prevention or intervention, and clues to the cause of the disease. Both genetic and environmental factors have been implicated in the development of AD (Kawas and Katzman 1999), but the cause of AD remains unknown, and no cure or universally effective treatment has yet been developed.

Alcohol consumption is one possible risk factor for AD. Alcoholism is associated with extensive cognitive problems (Evert and Oscar-Berman 1995), including alcoholic dementia (Smith and Atkinson 1997). Because alcohol’s effects on cognition, brain disorders, and brain chemistry share some features with AD’s effects on these three areas, it is plausible that alcohol use might also increase the risk of developing AD (Tyas 1996). Investigating whether and to what degree alcohol use is related to AD is made more difficult by the challenges of diagnosing and distinguishing alcoholic dementia and AD. Such studies are important, however, because alcohol use is a common but preventable exposure, an association between alcohol and AD is biologically plausible, and knowledge of the effect of alcohol on AD may provide clues to the cause of AD.

This article briefly reviews biological evidence suggesting that alcohol use may be associated with AD. It also focuses on the evidence from epidemiologic studies that link people’s consumption of alcohol to whether they develop AD, considers the influence of tobacco use on the relationship between alcohol use and AD, and examines the epidemiologic evidence of the connection between alcohol consumption and types of cognitive impairment other than AD.

## Effects of Alcohol Use on Brain Disorders and Cognition

Heavy alcohol consumption has both immediate and long-term detrimental effects on the brain and neuropsychological functioning (Delin and Lee 1992; Evert and Oscar-Berman 1995). Heavy drinking accelerates shrinkage, or atrophy, of the brain, which in turn is a critical determinant of neurodegenerative changes and cognitive decline in aging (Meyer et al. 1998). The shrinkage of brain tissue seen in Alzheimer’s disease and alcoholism is shown in the figure.

Changes observed with alcohol-related brain disorders, however, may be no more than superficially similar to those seen with aging or AD. In contrast to aging and AD, alcohol’s effects on the brain may be reversible (Carlen and Wilkinson 1987). Atrophy decreases after abstinence from alcohol (Kril and Halliday 1999). A study that further investigated cerebral atrophy in alcoholics and age-matched control subjects found no significant differences in the number of nerve cells in the brain (i.e., neurons) between the two groups and that most of the loss occurred in the white matter, which consists largely of nerve fibers that connect neurons (Jensen and Pakkenberg 1993). The researchers concluded that, because neurons did not appear to be lost, disrupted functions could be restored after abstinence as neuronal connections were reestablished. This conclusion is supported by research that also showed no neuronal loss in alcoholics compared with nonalcoholics but did show significant loss of brain cells that provide support for neurons (i.e., glial cells) which, in contrast to neurons, can be regenerated (Korbo 1999). That alcoholics can show improved cognitive performance after abstinence provides additional evidence of a reversible effect (Reed et al. 1992). Other studies, however, have reported neuronal loss with chronic alcohol abuse (Kril and Halliday 1999), including loss of neurons (i.e., cholinergic neurons) that contain or are stimulated by a certain chemical messenger in the brain (i.e., the neurotransmitter acetylcholine) (Arendt 1993). Cholinergic neurons are specifically affected in AD (see section below on biological mechanisms).

Improvement in cognitive function, or at least the lack of a progressive cognitive deficit, is one of the major factors used to determine whether a patient has alcoholic dementia rather than AD (Smith and Atkinson 1997). Recent work suggesting that characteristic neuropsychological profiles exist for alcoholic dementia and AD may prove useful in distinguishing the two disorders (Saxton et al. 2000). The diagnosis of alcoholic dementia, however, is itself somewhat controversial. Alcoholic dementia may have multiple causes. Pathological findings consistent with AD, nutritional deficiencies, trauma, and, in particular, stroke, also have been found in demented alcoholics (Fisman et al. 1996). The difficulty in distinguishing alcoholic dementia from AD has been attributed to a shared substrate of brain damage in the two disorders (Arendt 1993). A diagnosis of alcoholic dementia may be appropriate for some demented patients who have a history of alcohol abuse, but the effects of more moderate levels of drinking on cognitive function (for anyone) are not known. Thus, despite evidence of an association between alcohol use and neuropathologic and cognitive deficits, including alcoholic dementia, it is not yet clear whether alcohol use at either heavy or more moderate levels of consumption is associated with AD.

## Biological Mechanisms

Both alcohol and AD substantially affect the cholinergic system, and thus it is plausible that alcohol use could be linked to AD through their common effects on this system. Early studies of AD from the 1980s focused on the cholinergic system because it was known to play an important role in memory. Its role in AD was confirmed, and deficits in the cholinergic system, such as lower levels of acetylcholine and fewer receptors (proteins that bind to neurotransmitters), are now well established in AD. Although other neurotransmitter systems have since been implicated in AD, current treatment strategies still include repletion of cholinergic deficits (Forette and Boller 1999).

The cholinergic system also is affected by alcohol use. Chronic alcohol use causes degeneration of cholinergic neurons (Arendt 1993). Alcohol has been shown to decrease acetylcholine levels, reducing its synthesis and release. These deficits may aggravate the reductions already present in AD. Improvement of cognitive function in alcoholics after abstention from alcohol suggests that the cognitive deficits may reflect neurochemical alterations rather than neuronal loss (Kril and Halliday 1999). Alcohol-related memory loss can be partially reversed by compounds that stimulate the cholinergic system (e.g., nicotine; see section below on alcohol, tobacco, and AD) (Arendt 1993), illustrating the importance of the cholinergic system in alcohol’s effects on memory. Alcohol-induced cholinergic receptor losses in alcoholics with AD may contribute to the clinical symptoms of dementia. Alcohol does not appear to accelerate the AD process but instead induces its effects on the cholinergic system, independent of the cholinergic deficits caused by AD (Freund and Ballinger 1992). In addition, alcohol has extensive effects on neurotransmitter systems other than the cholinergic system and may also affect AD through these pathways (Diamond and Gordon 1997).

Alcohol may interact with both the brain and the aging process. In rodents, for example, age-related impairments in learning and memory are aggravated by alcohol consumption (Freund 1982). Alcohol-related brain damage appears to differ in young and old alcoholics (Kril and Halliday 1999). Although it has been suggested that alcohol abuse may accelerate aging-related changes in the brain at any age and that older adults may be more vulnerable to alcohol’s effects and thus show more age-related cognitive changes, these hypotheses of premature aging have been questioned (Evert and Oscar-Berman 1995). Arendt (1993) has suggested that the degenerative changes associated with aging, chronic alcohol abuse, and AD are on a continuum and that they may be quantitatively different but not qualitatively so.

Although a link between alcohol use and AD is plausible, whether such a relationship does exist or what the characteristics of such an association would be has not yet been established. For example, alcohol might affect whether one developed AD, when one developed it, or the progression of AD once one had developed it. Observing no association between increased numbers of senile plaques (a characteristic marker of AD found in the brain) and alcohol-related receptor loss, Freund and Ballinger (1992) concluded that alcohol consumption did not appear to accelerate the AD process. Although it has been neither proven nor disproven that alcohol increases the risk of developing AD nor lowers the age at onset, this study suggests that alcohol does not appear to affect progression of the disease. This hypothesis is supported by a study that reported that past heavy alcohol consumption was not associated with progression of AD over a 1-year interval (Rosen et al. 1993). Other researchers, however, have found past or current alcohol abuse to be a significant predictor of rate of decline in AD (e.g., Teri et al. 1990).

## Epidemiologic Studies of Alcohol Use and Alzheimer’s Disease

Many studies have examined the effects of alcohol and alcoholism on cognitive function and the brain. However, relatively few epidemiologic studies have focused on whether people who drink alcohol have a greater or lesser chance of developing AD. These studies are described here and summarized in the table.

Epidemiologic studies of alcohol use and AD in the 1980s and early 1990s generally were based on a case-control design, which identifies people with AD (i.e., cases) and a corresponding group of people without AD (i.e., control subjects) and then investigates whether alcohol consumption differs between these two groups. Relatively quick and inexpensive, the case-control design is a standard epidemiologic approach used to identify potential risk factors and to determine whether more extensive studies are warranted.

A summary of 11 of these case-control studies showed that 9 of the studies found no significant relationship between alcohol use and AD, 1 found that alcohol use increased the risk, and 1 found that alcohol use decreased the risk of AD (Tyas 1996). Most of these studies examined drinking status of study participants (whether they consumed alcohol at a specific, usually high level) rather than using more detailed measures of amount consumed. These case-control studies, however, may not have found a significant association because they had too few subjects (often less than 100 cases) and thus lacked statistical power. This possibility was addressed in two reports from a meta-analysis (Graves et al. 1991; van Duijn and Hofman 1992) that pooled the data from four individual case-control studies. However, the researchers did not find significant results for low, moderate, or high alcohol consumption even with this larger sample. Graves and colleagues (1991) conducted another meta-analysis that included a fifth study, which had used a different definition of alcohol use, but they still did not find a significant association between alcohol use and AD. Meta-analyses have increased power to detect significant associations but are still limited by the flaws of their constituent individual studies.

Subsequent case-control (Wang et al. 1997) and cross-sectional studies (Callahan et al. 1996) also have failed to provide evidence of an association between alcohol use and AD. One case-control study that did find a significant effect reported a reduced risk of AD in men with “high” alcohol use (i.e., more than two drinks per day), taking into account smoking status, education, and the status of a genetic marker for AD (apolipoprotein E allele, a variant of a gene) (Cupples et al. 2000).

Although the weight of evidence from the studies summarized above suggests that alcohol use is not related to AD, any conclusions must take into account the methodological limitations of these types of studies. The early studies often failed to account for confounding factors; drinkers differ from nondrinkers in many characteristics such as tobacco use and educational level and it may be those characteristics that are related to the risk of AD rather than alcohol use per se.

The case-control design also has inherent limitations. One notable weakness is that it is essentially cross-sectional. Longitudinal studies collect data on alcohol use at baseline and follow study participants over time to determine if they develop AD. Because high levels of alcohol use are associated with greater mortality, drinkers may be more likely than nondrinkers to die before developing AD, so a protective association between alcohol use and AD may simply reflect selective mortality. Clearly, longitudinal studies provide a better design from which to address issues such as selective mortality.

In addition, case-control studies collect information on alcohol use after diagnosis of AD. But because the cognitive deficits characteristic of AD mean that self-reported information cannot be obtained from study participants, proxy respondents (e.g., family members) are required. A proxy’s report is unlikely to correspond perfectly with the information that the study respondent would have provided. This problem is exacerbated if this source of error is not consistent across cases and controls (i.e., studies that use proxy reports for cases should also use proxy reports for controls). A methodological flaw in some of the case-control studies of AD (e.g., Cupples et al. 2000) has been the use of proxy-reported information for cases but self-reported data for controls.

Because of the methodological limitations of case-control studies, evidence from cohort studies—a stronger, longitudinal design—is usually given more weight, even though they also may have limitations (e.g., determination of AD based on clinical records rather than personal examination as per standard diagnostic criteria [Räihä et al. 1998]). Cohort studies generally have found no significant effect of alcohol use on the risk of developing AD (Brayne et al. 1998; Broe et al. 1998; Hebert et al. 1992; Katzman et al. 1989; Räihä et al. 1998; Tyas et al. 2001; Yoshitake et al. 1995), although some evidence of a protective effect of moderate wine consumption (defined as three to four glasses per day) has been reported—that is, moderate wine consumption has been associated with a decreased risk for AD (Leibovici et al. 1999; Orgogozo et al. 1997).

It is reasonable to expect that the effect of alcohol use on the risk of developing AD might differ depending on the level of alcohol consumption studied, but this does not seem to explain the study results. The cohort studies that found no association between alcohol use and AD used a variety of measures of alcohol consumption, from drinking status (Yoshitake et al. 1995), to amount of alcohol consumed (Broe et al. 1998), to alcohol abuse (Brayne et al. 1998). Both studies reporting protective effects (Leibovici et al. 1999; Orgogozo et al. 1997) were based in France and focused on wine consumption. It is possible that a protective effect is specific to this situation—that wine rather than other types of alcohol, in the drinking pattern and context of French culture, could be protective. In the study by Leibovici and colleagues (1999), however, the decreased risk of AD with alcohol use was reversed to become a significantly increased risk when the participants’ place of residence was considered (i.e., in the community or in an institution). Specifically, moderate wine consumption was associated with a lower risk of AD when place of residence was not considered, but with an increased risk when it was included in the analyses. In addition, the significant protective effect of moderate wine consumption reported in the French longitudinal study (Orgogozo et al. 1997) was based on very few cases of AD. Although overall most epidemiologic studies, regardless of the design, do not support an association between alcohol use and AD, further longitudinal studies are needed that overcome the methodological limitations of previous studies. The apparent lack of association between alcohol use and AD in epidemiological studies contrasts with alcohol’s proven effects on cognition, neuropathology, and neurochemistry, and its association with dementias other than AD. If it is determined that alcohol does influence the risk of AD, then understanding the mechanism by which it exerts this effect may provide clues to causal pathways, interventions, and prevention.

## Alcohol, Tobacco, and Alzheimer’s Disease

The effect of alcohol use on AD may be modified by other concurrent factors, such as tobacco use. Tobacco and alcohol use are related: “smokers drink and drinkers smoke” (National Institute on Alcohol Abuse and Alcoholism [NIAAA] 1998). The heaviest drinkers are the most likely to smoke, and 70 percent to almost 100 percent of alcoholics in treatment programs report smoking (Bobo and Husten 2000; NIAAA 1998). Conversely, a smoker is 10 times more likely than a nonsmoker to become an alcoholic (NIAAA 1998).

The prevalence of concurrent alcohol and tobacco dependence suggests that alcohol and tobacco may share mechanisms that lead to dependence (Anthony and Echeagaray-Wagner 2000). These mechanisms may have a genetic basis (Madden et al. 2000). Tobacco and alcohol use may be related at least partially because both nicotine and alcohol affect brain nicotinic cholinergic receptors (Collins 1990; Little 2000). Stimulation of these receptors is thought to contribute to the therapeutic effects of galantamine (Reminyl), a new treatment for AD (Maelicke et al. 2001; Newhouse et al. 2001).

Research shows that alcohol and tobacco use interact to influence the risk of certain diseases, such as cancer (U.S. Department of Health and Human Services 1989). Nicotine counteracts some of alcohol’s negative effects on cognition, including increased reaction time, impaired time judgment, and slowing of brain wave activity (Arendt 1993). Epidemiologic studies have begun to investigate the effect of an interaction between smoking and drinking on AD. Adjusting for smoking status had little effect on the association between alcohol use and AD in the case-control study by Cupples and colleagues (2000).

However, an analysis of three case-control data sets (Tyas et al. 2000) has provided some support for the hypothesis that smoking influences the effect of alcohol use on AD. In one of the data sets, the risk of AD was significantly increased in drinkers. Study participants who smoked as well as drank, however, had a lower risk than those who only drank. The pattern in the other two data sets varied depending on whether the participants had a history of hypertension. A pattern similar to that of the first data set, but only marginally significant, was found for hypertensive subjects in a second data set, with the risk of AD for people who were both smokers and drinkers lower than the risk for those who were just smokers or just drinkers. It is not clear whether the effect of hypertension reflects a physiological interaction of hypertension with smoking, drinking, and AD. Few analyses on the interaction of tobacco and alcohol use have been published, but one study (Brenner et al. 1993) did find that the association between smoking and AD varied by the hypertensive status of study participants.

The observation that alcohol and tobacco use appear to influence each other’s association with AD is consistent with evidence of a biological interaction between smoking and drinking. This observation also may be attributed, however, to the increased overall mortality of people who both smoke and drink, a possibility that can only be ruled out by longitudinal research. The apparent importance of hypertension suggests that a vascular mechanism may be involved in the interaction of alcohol and tobacco use on the risk of developing AD.

## Epidemiologic Studies of Alcohol Use and Cognitive Impairment

Although epidemiologic studies do not generally support an association between alcohol consumption and AD, the lack of such a relationship could reflect methodological limitations, such as the difficulty in discriminating AD cases with a history of heavy alcohol consumption from cases of alcoholic dementia. It thus may also be useful to consider evidence from epidemiologic studies examining the association between alcohol use and cognitive outcomes other than AD. The ways in which alcohol use influences the risk of developing cognitive impairment might be similar to those by which it may affect AD, and some types of cognitive impairment themselves may increase the risk of developing AD.

Overall, the results of epidemiologic studies of alcohol use and cognitive impairment are consistent with results from studies of alcohol use and AD. Most studies, regardless of design, found no significant association between alcohol use and cognitive impairment (Cervilla et al. 2000*b*; Christian et al. 1995; Dent et al. 1997; DiCarlo et al. 2000; Dufouil et al. 2000; Edelstein et al. 1998; Elwood et al. 1999; Hebert et al. 1993). Those studies which did report a significant effect of alcohol use generally found that the results varied by gender (Dufouil et al. 1997), by apolipoprotein E allele status (Carmelli et al. 1999; Dufouil et al. 2000), or by vascular risk factors (e.g., cardiovascular disease and diabetes) (Launer et al. 1996). Evidence of these subgroup effects is not yet compelling; for example, in people with the apolipoprotein E allele, alcohol use increased the risk of cognitive impairment in one study (Dufouil et al. 2000) but decreased it in another (Carmelli et al. 1999). In another study, apolipoprotein status had no effect (Cervilla et al. 2000*a*). However, investigation of the effects of alcohol use on AD within these gender, genetic, or vascular risk subgroups may prove informative.

## Does Alcohol Use Cause Alzheimer’s Disease?

Although an increased risk of AD with alcohol use is plausible based on biological evidence, the epidemiologic evidence does not support an association. In the few studies that report a significant association, alcohol consumption is more often found to reduce the risk of AD than to increase it. However, methodological factors could create an apparent protective effect of alcohol use on AD. Such factors include selective mortality of drinkers and diagnosing AD patients with heavy alcohol use as having alcoholic dementia rather than AD. In addition, in some studies reporting a protective effect of alcohol (e.g., Cupples et al. 2000), proxy respondents provided information for the cases whereas self-reported information was used for controls. If proxy reports of drinking underestimate actual exposure (McLaughlin et al. 1990), the alcohol use of cases (i.e., study participants with AD) would be artificially lowered compared with control subjects. The apparent association between alcohol use and a reduced risk of AD might therefore merely reflect bias in proxy reports rather than any true effect. Other recent reports of a protective effect (Orgogozo et al. 1997; Leibovici et al. 1999) may have been affected by sample size and the selection of confounding factors, such as community or institutional residence, included in the analyses.

Most studies, including the meta-analysis of case-control studies (Graves et al. 1991; van Duijn and Hofman 1992) and individual cohort studies of AD (Brayne et al. 1998; Broe et al. 1998; Hebert et al. 1992; Katzman et al. 1989; Räihä et al. 1998; Tyas et al. 2001; Yoshitake et al. 1995) have not found a significant association. Epidemiologic studies of alcohol use and cognitive impairment overall have come to similar conclusions, although some evidence exists for a heterogeneous effect of alcohol use on cognitive impairment across gender, genetic, or vascular subgroups.

The effect of alcohol use on the risk of AD has been explored much less extensively than the effect of other potential risk factors, such as tobacco use. The possibility of a protective effect of moderate drinking on AD, raised in a few studies, may not be compelling, but methodological issues need to be resolved before such an association can be definitively dismissed. Moderate drinking has been reported to have some beneficial vascular effects (NIAAA 2000), which could possibly reduce the risk of AD. The nonsignificant association between alcohol use and risk of AD reported by most studies does not necessarily mean that alcohol has no effect. It may instead reflect a balance between the beneficial vascular effects of alcohol and its detrimental effects on the brain, and the relative weight of these two factors may differ within specific subgroups.

## Limitations of Current Studies

Because AD has few established risk factors, most studies have examined alcohol use as only one possibly relevant exposure among many, necessitating superficial treatment. Future studies need to collect more detailed information about lifetime alcohol exposure because imprecision in estimating lifetime exposure may obscure associations, as may inconsistent definitions of drinking status or level of consumption. Evidence that alcohol’s effects on AD might vary within subgroups also supports more extensive data collection on variables that characterize these subgroups.

One methodological challenge of both case-control and cohort studies is the separation of AD from alcoholic dementia. AD cannot be definitively diagnosed clinically but instead requires confirmation based on examination of the brain after death. Even when AD is accurately diagnosed before death, study participants still represent a heterogeneous group, differing in age at onset, duration, and genetic basis of AD. Case-control studies may introduce bias by using heavy alcohol consumption as an exclusionary criterion for AD cases but not for controls (e.g., Graves et al. 1991). As alcoholic dementia has not been uniformly diagnosed across epidemiologic studies, the discrimination of alcoholic dementia from AD also is problematic.

## Priorities for Future Research

Further longitudinal studies that overcome the methodological limitations described above are needed to address basic issues such as selective survival bias. Better measures of lifetime alcohol exposure will help delineate critical exposure periods. Fundamental questions on the biological association between alcohol and AD remain unanswered, such as whether alcohol is associated with pathological and pharmacological changes characteristic of AD. Research is needed to clarify the effects of alcohol on cognitive abilities and the mechanisms by which alcohol may act to influence the risk of developing AD. Additional priority areas include research into the possibility of concurrent detrimental and beneficial effects of alcohol on AD across all levels of alcohol consumption. Investigating the effect of alcohol use within gender, genetic, vascular, or other subgroups might reveal associations with AD more clearly.

## Conclusions

Although it is biologically plausible that drinking increases the risk of AD, epidemiologic studies have not supported this hypothesis. Currently, no strong evidence suggests that alcohol use influences the risk of developing AD, but further research is needed before the effect of alcohol use on AD is fully understood.

## Figures and Tables

**Figure:**
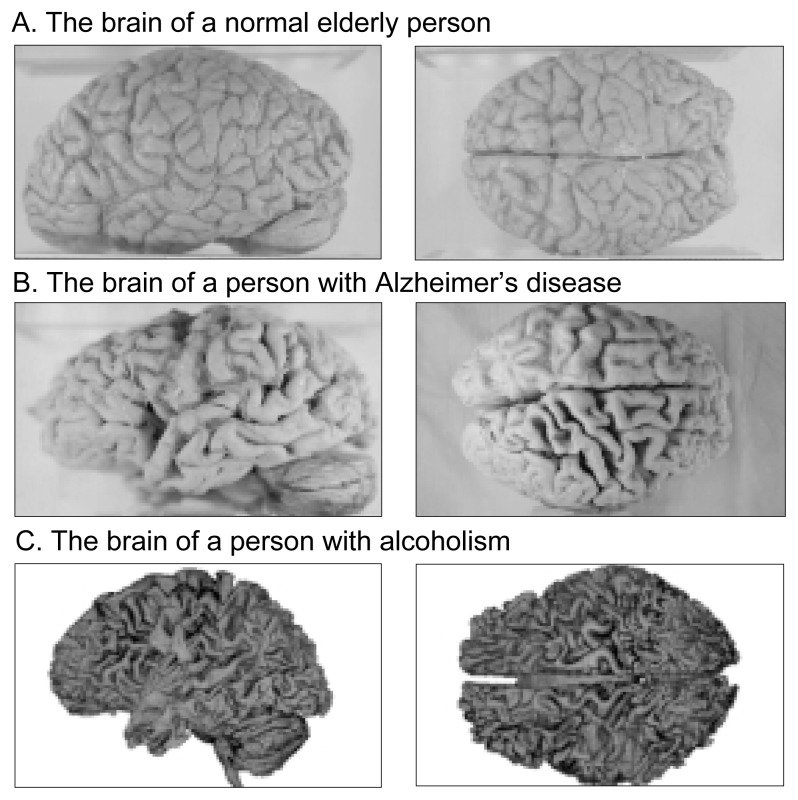
Compared with the brain of a normal elderly individual (Panel A), the wider grooves and narrower ridges of the brains in Panels B and C reflect the shrinkage of brain tissue seen in Alzheimer’s disease and alcoholism. SOURCE: Photographs in panels A and B courtesy of Sanders-Brown Center on Aging, University of Kentucky. Panel C originally appeared in *Alcohol Health & Research World* 19(4), 1995, p. 268.

**Table:** Summary of Major Epidemiologic Studies of Alcohol Use and the Risk of Developing Alzheimer’s Disease (AD) by Type of Study Design

Study Design	Alcohol use increased the risk of AD	Alcohol use decreased the risk of AD	Alcohol use had no significant effect on the risk of AD
Cross-sectional	0	0	1
Case-control	1	2	10
Cohort	0	2	7
Meta-analysis	0	0	1
**Total**	**1**	**4**	**19**

*Cross-sectional* studies provide a snapshot of a disease (e.g., AD) at a single point in time and examine relationships between the disease and other factors, such as alcohol use.

*Case-control* studies of alcohol use and AD compare people with AD (cases) with people without AD (controls) and determine whether alcohol consumption differs between the two groups.

*Cohort* studies provide a stronger, longitudinal design (i.e., they collect data on alcohol use at baseline and follow study participants over time to determine whether they will develop AD).

A *meta-analysis* pools data from multiple studies and thereby offers increased statistical power.
